# Mechanisms of adhesive small bowel obstruction and outcome of surgery; a population-based study

**DOI:** 10.1186/s12893-020-00724-9

**Published:** 2020-04-06

**Authors:** Thorbjörn Sakari, Malin Christersson, Urban Karlbom

**Affiliations:** 1 Department of Surgical Sciences, Uppsala University, Gävle Hospital, SE-803 24 Gävle, Sweden; 2 Department of Surgical Sciences, Uppsala University, University Hospital, Uppsala, Sweden

**Keywords:** Small bowel obstruction, Adhesions, Emergency surgery, Bowel resection, Recurrence, Surgery

## Abstract

**Background:**

This study aims to describe the mechanisms of adhesive small bowel obstruction (SBO) and its morbidity, mortality and recurrence after surgery for SBO in a defined population.

**Method:**

Retrospective study of 402 patients (240 women, median age 70 years, range 18–97) who underwent surgery for SBO in the Uppsala and Gävleborg regions in 2007–2012. Patients were followed to last note in medical records or death.

**Result:**

The cause of obstruction was a fibrous band in 56% and diffuse adhesions in 44%. Early overall postoperative morbidity was 48 and 10% required a re-operation. Complications, intensive care and early mortality (*n* = 21, 5.2%) were related to age (*p* < 0.05) and American Society of Anesthesiologist’s class (*p* < 0.01). At a median follow-up of 66 months (0–122), 72 patients (18%) had been re-admitted because of SBO; 26 of them underwent a re-operation. Previous laparotomies (*p* = 0.013), diffuse adhesions (*p* = 0.050), and difficult surgery (bowel injury, operation time and bleeding, *p* = 0.034–0.003) related to recurrent SBO. The cohort spent 6735 days in hospital due to SBO; 772 of these days were due to recurrent SBO. In all, 61% of the cohort was alive at last follow-up. Late mortality was related to malignancies, cardiovascular disease, and other chronic diseases.

**Conclusions:**

About half of patients with SBO are elderly with co-morbidities which predispose to postoperative complications and mortality. Diffuse adhesions, which make surgery difficult, were common and related to future SBO. Overall, nearly one-fifth of patients needed re-admission for recurrent SBO. Continued research for preventing SBO is desirable.

**Trial registration:**

The study was registered at ClinicalTrials.gov (NCT03534596, retrospectively registered, 2018-05-24).

## Background

Almost all patients will develop intra-abdominal adhesions after abdominal surgery [1–4]. The most common consequences are: more complex subsequent surgery, abdominal pain, small bowel obstruction (SBO), and infertility. A 35% readmission rate over 10 years after abdominal surgery has been reported to be directly or possibly related to adhesions [1]. The risk of developing SBO that requires surgery varies from 1% after appendectomy [5, 6] to more than 10% after colectomy [3, 7]. About 20% of those who do develop SBO do so within the first postoperative year [1, 2, 7]. Thereafter, there is a steady increase in prevalence for up to at least 10 years after the initial operation [8]. There are conflicting reports on the optimal timing of surgery for small bowel obstruction. Most studies advocate early surgery in line with the Bologna guidelines for SBO [9, 10], to minimise morbidity and mortality, although some take a more conservative approach [11].

There is a limited number of reports on direct outcome measures after SBO surgery. The studies that do exist involve relatively few patients [12] or focus on older material [13, 14]. Thus, studies analysing the clinical course after surgery for SBO in larger cohorts in more recent time periods are needed.

The aim of this study was to describe the mechanisms of adhesive SBO, as well as its morbidity, mortality, and recurrence after surgery for SBO in a defined population.

## Methods

This study included adult patients operated on for adhesive SBO in the Uppsala and Gävleborg regions. There were 341,977 inhabitants in Uppsala and 276,637 in Gävleborg, which together make up 6.5% of the Swedish population. Emergency surgery was performed in three hospitals in the regions (Gävle County Hospital, Hudiksvall Hospital and Uppsala University Hospital). The time period (1 Jan 2007 to 31 Dec 2011) was selected to get a cohort of sufficient size and a follow-up of at least 5 years.

**Fig. 1:**
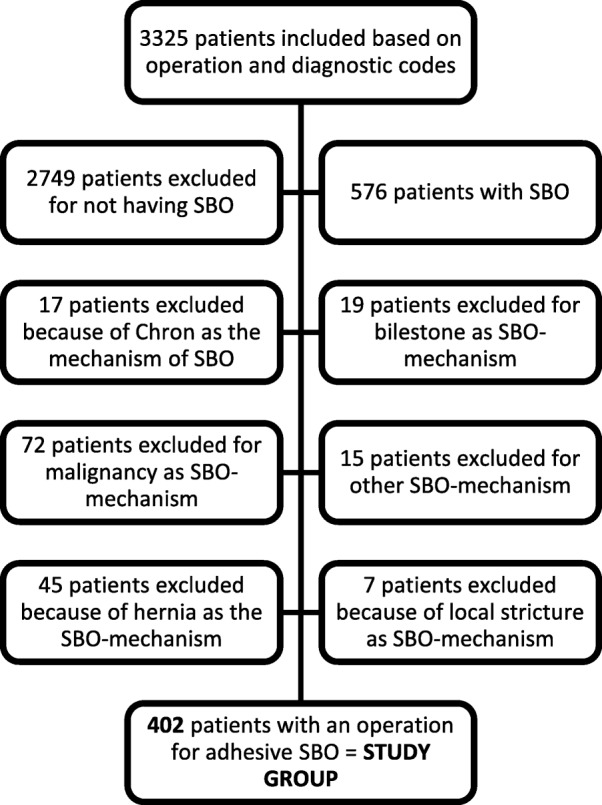
Overview diagram of inclusions and exclusions in the study population

A search for adult patients (≥ 18 years) possibly operated on for SBO was performed using operation and diagnostic codes (R10, K56, JAH, JAP, JFB, JFK, and JFL) in the computer-based medical record systems. Patients with specific causes for obstruction, other than adhesions, were excluded, whit the remainder making up the study group (Fig. 1). Medical records were then analysed based on a protocol. Co-morbidities, type procedure, and number of previous operations were noted. The obstructive mechanisms, as described in the operation charts, were noted and classified as fibrous bands or diffuse adhesions. Where there was a description of a combination of bands and diffuse adhesions, a closer analysis of the operation chart was performed to determine whether bands or diffuse adhesions were the main cause of obstruction.

The following short-term complications (≤30 days) were analysed: anastomotic leak (diagnosis with contrast enema, CT or re-operation), abscess (diagnosis with CT or ultrasound), wound infection, wound dehiscence, cardiovascular, pulmonary, and urinary tract infection (positive culture). Cardiovascular complications included myocardial infarction and arrythmias, while pulmonary complications included aspiration and pneumonia. ICU stays, re-operations and mortality were also noted. Complications within 30- days were classified based on Clavien-, grades 2–5 [15]. Analysis of long-term complications (> 30 days) was focused on hospitalisation and surgery for recurrent SBO (SBO defined as presence of at least two of the following symptoms: loss of stool or flatus, nausea/vomiting, abdominal distension, and radiology supporting SBO). Incisional hernia was noted if mentioned in the charts or in the radiological report.

Data from medical records were collected until 2017. Follow-up time was calculated from the SBO- operation to the last noted contact in records or death. The study was approved by the local ethical committee at Uppsala University (Dnr 2015/196) and registered at ClinicalTrials.gov (NCT03534596, 2018-05-24).

### Surgery

The three hospitals had similar clinical routines, but there was no written protocol for management of SBO. Patients without signs of strangulation were initially treated with nasogastric tube, intravenous fluids and analgesics. Patients with signs of strangulation underwent urgent surgery; the others were subjected to radiology. The surgeon managing the patient made the decision to operate and determined the timing of the operation, based on his or her judgement. Laparoscopic surgery for SBO was not in practice during the study period, so all patients had open surgery through midline incisions. Data were collected on type of procedure (division of band, adhesiolysis, bowel resection, by- pass, or stoma), anastomoses, bowel- injuries, bleeding, and operating time.

### Statistical analysis

Demographic and clinical data were analysed using χ2 tests for categorical variables and t-tests for continuous variables or Mann-Whitney U-tests in the case of non-normal distributions. Fisher’s exact test was used instead of the χ2 test when low expected counts were observed. Numbers are given as means and ranges unless otherwise stated. Data were analysed using the statistical package SPSS® version 25 for Windows® (SPSS, Chicago, IL, USA). A two-sided *P* value ≤0.05 was considered to be statistically significant. No power calculation was made.

## Results

### Patients

The search of medical records resulted in 3326 patients possibly operated for SBO. After exclusions, a study group of 402 patients was identified (Fig. 1). In all, 240 (60%) were women and 162 (40%) were men. The median age was 70 years (range 18–97 years). More than half of patients had some co-morbidity (Table 1) with the most common being cardiovascular disease (hypertension and other arterial disease, cardiac disease, cerebro- vascular disease). The preoperative American Society of Anesthesiologist’s (ASA) classification was 3 or 4 for 48% (Table 1). Thirty-seven patients (9%) had a previous malignant abdominal disease, with 23 of them having undergone radiation therapy to the pelvis as part of cancer treatment (rectal, prostate, or gynaecological cancer). Abdominoperineal resection had been performed in seven, all of whom had undergone radiation therapy. There were no signs of recurrent cancer as a cause of recurrent SBO.

**Table 1:** Demographic data and co-morbidity

	Patients ***n*** = 402
Gender M/F	162/240 (40%/60%)
Age, in years, median, (range)	70 (18–97)
< 40 years	37 (9%)
> 80 years	99 (25%)
Co- morbidity	
ASA 1–2	204 (51%)
ASA 3–4	193 (48%)
MD^a^	5 (1%)
Cardiovascular	202 (50%)
COPD^b^	45 (11%)
Diabetes	36 (9%)
Immunosuppression	25 (6%)
Any of the above	232 (58%)

^a^missing diagnoses, ^b^Chronic Obstructive Pulmonary Disease

Fifty patients (12%) had not undergone any abdominal surgery before the index operation whereas 189 (47%) had one previous abdominal operation (Table 2). Nineteen of those with one previous abdominal operation had had it done laparoscopically, one (5%) of them later developed SBO and was admitted for 2 days of conservative management. Previous surgery for SBO had been performed in 30 patients (7.5%). There were no differences in demographic data or previous surgery between the two regions.

**Table 2:** Previous abdominal surgery in relation to index operation for SBO

No previous abdominal operations	Women ***n*** = 240	Men ***n*** = 162	All n = 402
0	22 (9%)	28 (17%)	50 (12%)
1	106 (44%)	83 (51%)	189 (47%)
2	46 (19%)	35 (22%)	81 (20%)
3	47 (20%)	13 (8%)	60 (15%)
≥4	19 (8%)	3 (2%)	22 (6%)
Type of procedure*			
Appendectomy	80 (33%)	35 (22%)	115 (29%)
Colorectal	60 (25%)	50 (31%)	110 (27%)
Gynaecological	106 (44%)	0 (0%)	106 (26%)
Upper GI	69 (29%)	35 (22%)	104 (26%)
Surgery for SBO	17 (7%)	13 (8%)	30 (7%)
**Previous surgery within**	*n* = 218	*n* = 134	*n* = 352
1 year	47 (22%)	36 (27%)	83 (24%)
1–2 years	8 (4%)	9 (7%)	17 (5%)
2–5 years	22 (10%)	18 (13%)	40 (11%)
> 5 years	141 (65%)	71 (53%)	212 (60%)

*Many patients had several operations, meaning that the total exceeds 100%

### Surgery

The mean annual number of SBO operation was 116 during the study period, adhesive SBO was the dominating cause, leading to 80 operations per year. Adhesive SBO surgery was performed on 13 per 100, 000 inhabitants and year and a total of 5963 days were recorded for hospital stays related to index surgery for SBO.

Almost all patients (391/402, 97%) underwent a CT- scan and most also had a contrast follow-through. All but one of the patients had some diagnostic radiology before surgery.

Nearly all patients (*n* = 384, 96%) underwent surgery after being admitted to the emergency department, but 18 (5%) patients underwent surgery in the postoperative phase after other abdominal surgery. Overall, patients were operated on at mean 3.4 days (0–86) after hospitalisation or SBO- diagnosis. A total of 78 (19%) patients underwent an operation on the day of admission and another 120 (30%) were operated on within 2 days of hospitalisation.

Delaying surgery more than 2 days after admission was related to more re-operations (15% vs. 8%, *p* = 0.046) compared to those operated in the first 2 days. Similarly, surgery later than 4 days after admission was related to more re-operations (17% vs. 9%, *p* = 0.024) and more wound dehiscence (9% vs. 4%, *p* = 0.037). Beyond that, the timing of surgery was not related to mortality, short- or long-term complications, or bowel resection frequency. Nearly one-third (*n* = 122) had a period of conservative treatment longer than 3 days. Bowel injury was common, see Table 3.

**Table 3:** Bowel injury related to the number of previous laparotomies

	Bowel injury	No bowel injury
Total n = 402	153 (38%)	249 (62%)
No. previous abdominal surgeries		
0 (*n* = 50)	7 (14%)	43 (86%)
1 (*n* = 189)	67 (35%)	122 (65%)
2 (*n* = 81)	40 (49%)	41 (51%)
3 (*n* = 60)	29 (48%)	31 (52%)
≥ 4 (n = 22)	10 (45%)	12 (55%)
Intraabdominal abscess	22/153 (14%)	22/249 (9%)
Enterocutaneous fistula	2 (1.3%)	0

In all, 139 patients (35%) had bowel resections (137 small bowel resections and 5 ileocecal resections, some had both). The resection length varied from 2 to 300 cm. Anastomotic leak was noted in 4 of the 139 (2.9%) patients with an anastomosis. In another eight patients, a by-pass was performed, most often for dense diffuse adhesions or due to difficult anatomy.

### Adhesive mechanisms

The most common obstructing mechanism was a fibrous band, which was found in 226 (56%) of the patients. This was also the dominating cause for SBO in patients without a history of laparotomy (44/50, 88%). Patients with diffuse adhesions had more co-morbidity, more previous abdominal surgery, and a longer period of conservative treatment before being operated on, compared with patients with band adhesions (Table 4). A longer operation time, more bleeding and more bowel injuries were observed in the diffuse adhesion group (Table 4).

**Table 4:** Preoperative data, early and long-term complications in relation to band or diffuse adhesions as the mechanism of SBO

	Band adhesion ***n*** = 226	Diffuse adhesions ***n*** = 176	*p*- value
Age (years)^b^	66.7 ± 18	66.8 ± 17	0.949
Gender m/f	83/143	79/97	0.098
ASA 1–2^a^	125 (55%)	79 (45%)	0.027
ASA 3–4	97 (44%)	96 (55%)	0.027
No. previous operations^b^	1.3 ± 1.1	1.8 ± 1.1	< 0.001
Admission to surgery (days, mean)^b^	2.5 ± 5.2	4.5 ± 8.5	< 0.001
Bleeding (ml)^b^	106 ± 194	304 ± 447	< 0.001
Operation time (min)^b^	75 ± 43	122 ± 74	< 0.001
Bowel injury	50 (22%)	103 (59%)	< 0.001
Any bowel resection	71 (31%)	68 (39%)	0.131
By-pass	1 (0.4%)	7 (4%)	0.012
**Early complications**	99 (44%)	92 (52%)	0.092
Anastomotic leak (*n* = 139)	0/71 (0%)	4/68 (7%)	0.055
Wound dehiscence	7 (3%)	12 (7%)	0.081
Wound infection	9 (4%)	12 (7%)	0.205
Intraabdominal infection	21 (9%)	23(13%)	0.229
Aspiration	8 (4%)	7 (4%)	0.818
Pneumonia	21 (9%)	11(6%)	0.264
Myocardial infarction	3 (1%)	4 (2%)	0.472
Arrythmia	12 (5%)	9 (5%)	0.930
Urinary tract infection	19 (8%)	13 (7%)	0.708
Clavien-Dindo 2	59 (26%)	45 (26%)	0.903
Clavien-Dindo 3	12 (5%)	13 (7%)	0.392
Clavien-Dindo 4	18 (8%)	22 (12%)	0.132
Clavien-Dindo 5	11 (5%)	10 (6%)	0.716
Postoperative stay (days)^b^	8.7 ± 7.1	13.6 ± 13.2	< 0.001
Length of stay (days)^b^	11.5 ± 7.7	19.1 ± 16.7	< 0.001
**Late complications**			
SBO- admission	33 (15%)	39 (22%)	0.050
SBO- admission days (mean)^b^	1.9 ± 4.1	5.6 ± 11.9	0.002
No. times admission for SBO (range)	0.44 (0–3)	1.02 (0–12)	0.004
SBO- surgery	13 (5.8%)	13 (7.4%)	0.509
Hernia	11 (5%)	13 (7%)	0.290
Deceased at follow-up	73 (32%)	85 (48%)	0.001

^a^ASA- class: 5 patients missing. ^b^Mean ± SD.

The postoperative stay and total length of stay were significantly longer in the diffuse adhesion group. Anastomotic leaks, and unspecified use of antibiotics were more frequent in this group, and more of these patients died during follow-up (Table 4). Furthermore, diffuse adhesions were related to more admissions for SBO and longer hospital stay during follow-up, but not to surgery for SBO.

### Early complications (≤ 30 days)

One hundred and ninety-one (48%) patients had an early postoperative complication (Table 5). The most common complication was intra-abdominal infection which affected 44 (11%) patients. Age over 70 years was related to increased complication frequency (Table 5). Neither gender nor number of previous laparotomies affected complications (data not shown). Patients with ASA- class 3–4 had a complication frequency of 122/193 (63%) compared with 69/204 (34%) for ASA- class 1–2 (*p* < 0.001).

**Table 5:** Early and late complications in relation to age at surgery

	Age < 70 years *n* = 201	Age ≥ 70 years n = 201	p- value	All *n* = 402
**Early**				
Anastomotic leak (*n* = 139)	4/66 (6.1%)	0/73(0.0%)	0.048	4/139 (2.9%)
Wound dehiscence	4 (2.0%)	15 (7.5%)	0.010	19 (4.7%)
Wound infection	14 (7.0%)	7 (3.5%)	0.117	21 (5.2%)
Intraabdominal infection	21 (10.4%)	23 (11.4%)	0.749	44 (10.9%)
Aspiration	5 (2.5%)	10 (5.0%)	0.188	15 (32.7%)
Pneumonia	9 (4.5%)	23 (11.4%)	0.010	32 (8.0%)
Myocardial infarction	0 (0.0%)	7 (3.5%)	0.015	7 (1.7%)
Arrythmia	1 (0.5%)	20 (10.0%)	< 0.001	21 (5.2%)
Urinary tract infection	11 (5.5%)	21 (10.4%)	0.065	32 (8.0%)
Re-operation	18 (9.0%)	23 (11.4%)	0.410	41 (10.2%)
Intensive care	16 (8.0%)	35 (17.4%)	0.004	51 (12.7%)
ICU days^a^	0.62 ± 3.5	0.91 ± 3.7	0.433	0.76 ± 3.6
Postoperative stay (days)^a^	10.2 ± 10.8	11.4 ± 10.3	0.241	10.8 ± 10.5
**Late**				
SBO-admission	45 (22.4%)	27 (13.4%)	0.019	72 (17.9%)
No. times admission for SBO^a^	0.9 ± 1.9	0.5 ± 0.9	0.032	0.7 ± 1.5
SBO- admission days^a^	4.3 ± 9.8	2.5 ± 6.6	0.117	3.5 ± 8.5
SBO- surgery	17 (8.5%)	9 (4.5%)	0.105	26 (6.5%)
Hernia	15 (7.5%)	9 (4.5%)	0.207	24 (6.0%)
Deceased at follow-up	32 (16%)	126 (63%)	< 0.001	158 (39%)

^a^Mean ± SD

Forty-one (10%) patients had an early re-operation, including scheduled second look (*n* = 8), abdominal sepsis (*n* = 11), wound dehiscence (*n* = 13), recurrent/persistent SBO (*n* = 7), postoperative bleeding (n = 1), and severe abdominal pain (n = 1).

Fifty- one (13%) patients needed intensive care and they had an average stay of 6 (1–40) days.

### Late complications (> 30 days)

**Fig. 2:**
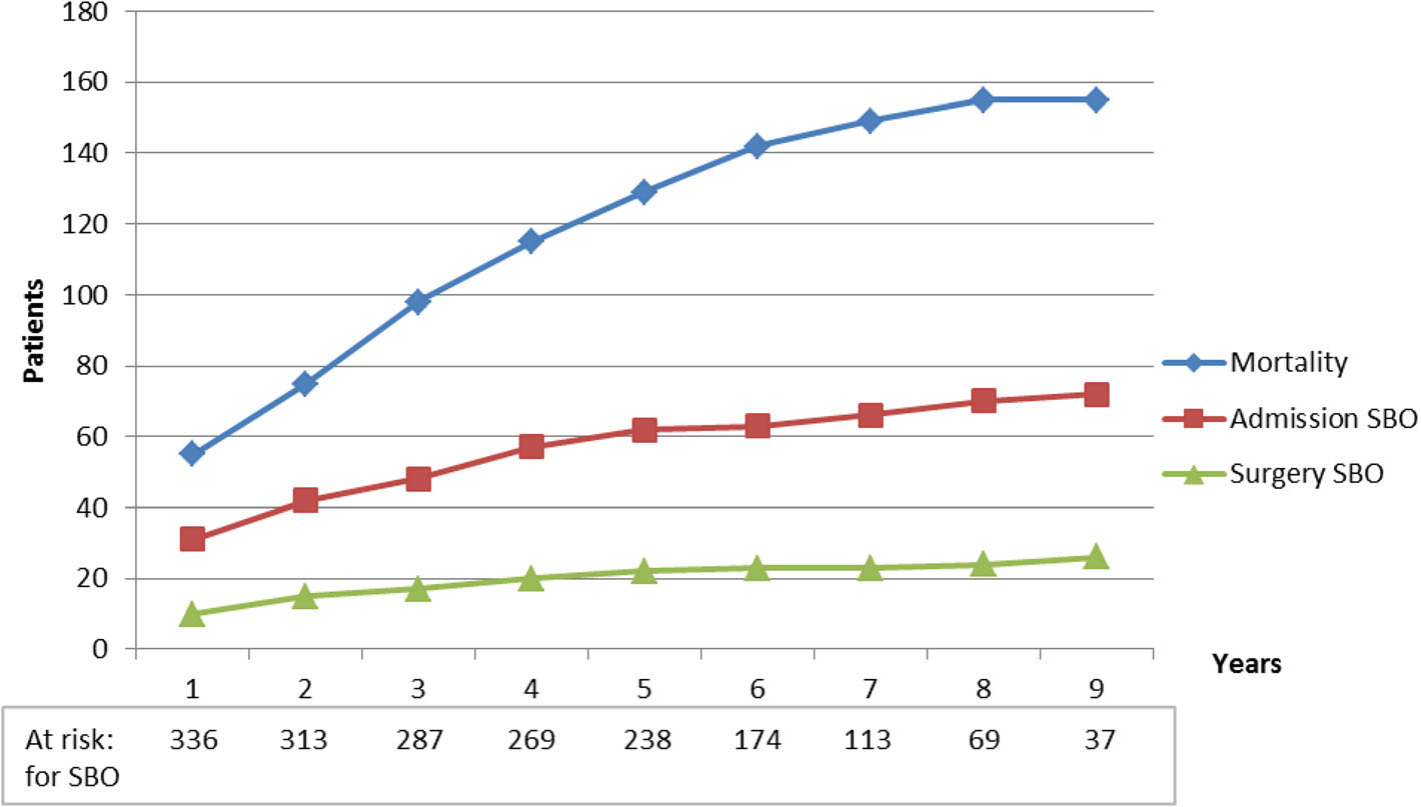
Cumulative data for overall mortality, re-admissions, and surgery for SBO

Median length of follow-up was 66 months (0–122). Total overall mortality was 39% (*n* = 158), with 21 patients dying in the 30-day postoperative period and 137 patients dying beyond 30 days (Fig. 2). The mean age for these patients at index surgery was 78 years and 83 of them (52%) were above 80 years. The majority (79%) were preoperatively classified as ASA 3–4. The most common causes of death were cardiovascular diseases, malignancies, and respiratory diseases. Median age at death was 83 (range 30–98) years. For three patients, the cause of death was clearly related to SBO: two died after surgery for recurrent SBO and one died during hospital stay and conservative treatment for SBO.

At follow-up, 72 patients (17.9%) had been admitted to hospital 1–12 times for symptoms of SBO. Of 201 patients below 70 years of age, 45 were admitted (22.4%) compared with 27 (13.4%) among patients above the age of 70 (*p* = 0.019). However, follow-up was longer for patients with recurrent SBO (median 74 vs. 64 months, *p* = 0.0014). Number of previous laparotomies (1.8 vs. 1.5, *p* = 0.013), bowel injury (39/72 vs. 114/330, *p* = 0.003), operating time (median 95 vs.78 min, *p* = 0.006) and peri operative bleeding (median 100 vs. 50 ml, *p* = 0.034) were related to recurrent hospitalisation for SBO. Twenty- six patients (6.5%) underwent another laparotomy because of SBO. Incisional hernia was confirmed in 24 (6%) of the patients during follow-up. Six (1.5%) patients underwent surgery because of incisional hernia during the follow-up period.

## Discussion

A striking finding in this study was the strong negative impact of diffuse adhesions causing an increased risk for complications, more complex surgery, a prolonged hospital stay, more future SBO episodes, and a shorter life span. In this study, diffuse adhesions were the cause of obstruction in nearly half of the patients. Adhesiolysis may be difficult in the case of diffuse adhesions: during surgery nearly 60% had bowel injuries and if resection was needed, there was a tendency for more anastomotic leaks. For individual patients, this might lead to more complications, but there were no differences overall in early complications compared with among patients with band adhesions. Fevang et al. found a 29% recurrence rate at 25 years, which rose to 40% in the subgroup with diffuse adhesions [16]. On average patients with diffuse adhesions had undergone more previous laparotomies and also had a higher ASA classification. Anticipated difficult surgery and/or, co-morbidity with risk of complications or death are possible reasons that these patients had a longer period of conservative treatment. In addition, there may have been other mechanisms of selection which cannot be assessed in a retrospective study. There were more women undergoing surgery for SBO, most likely reflecting previous gynaecological surgery. Gender did not affect other data or outcome. Demographic data also showed that many patients were old with co-morbidities; 25% of patients were older than 80 years and nearly all of them had co-morbidities resulting in ASA class 3–4. Surgery and postoperative care for this group is a challenge when trying to avoid complications and mortality.

Definitions of complications vary, and even missing in some studies, making direct comparisons difficult. Both complications and mortality are related to age which must be taken into account when comparing different studies. The current complication rate of 48% seems reasonable in view of the age distribution and co-morbidity. Thirty- day mortality was 5.2% (all being above 70 years) which is comparable to rates in other studies [13]. In this study, we had no increased mortality when bowel injury was present. Long-term mortality was unexpectedly high, but analysis of causes of death showed that all but three died from diseases not related to SBO (cardiovascular, respiratory, malignant disease). Age at death did not differ from that of the general population in Sweden.

There was a small inflow (*n* = 10) of patients from other regions during the study period. About one-third of these were specific referrals due to complicated co-morbidity (transplant and vascular). The outflow of patients could not be assessed, but empirically knowledge indicates that there is no systematic referral to other regions. Emergency surgery during holidays or travels would account for patients being operated on elsewhere. We think this number is small, meaning that data could be regarded as population-based.

Thirteen SBO operations per 100, 000 inhabitants and year were performed. This was, a somewhat higher frequency than previously reported by Kossi et al. and Tingstedt et al. [17, 18], but on the other hand, less than half of what Ray et al. and Ellis et al. [1, 19] reported. This discrepancy may be due to differences in study design and local traditions or selection mechanisms. Balancing between conservative treatment and surgery is difficult in patients without peritonitis or strangulation. An elderly patient with co-morbidity and not requiring emergency surgery will probably start with a conservative regimen to avoid the surgical trauma. In our study, about 20% of patients needed same- day surgery, which is about equal to the rate reported by Bauer et al. [20]. Overall mean time to surgery was 3.4 days (including outliers). A possible risk of prolonged conservative treatment is that increasingly extensive resections are necessary. Surgery after more than 4 days after admission was related to more re-operations and wound dehiscence, but not to resections, mortality, or other complications. Since most SBO episodes resolve with conservative treatment [21], the balance between conservative treatment and surgery and timing of surgery is best assessed in a prospective manner. The current recommendation of early surgery seems reasonable.

Recurrent SBO was 7% at 1 year and 18% overall during follow-up, a third of these patients required surgery. About half of the recurrences appeared within the first 2 years, but there was a cumulative increase during follow-up. In this cohort, the number of patients at risk of recurrent SBO was reduced by the high overall mortality. Patients younger than 70 years, with lower mortality, also had a higher incidence of recurrent SBO (22.4%). Therefore, prevention of adhesions at initial surgery and at secondary SBO surgery is desirable. Minimally invasive surgery has been associated with less formation of adhesions and recent reviews found a reduction of SBO events and surgery for SBO after laparoscopic colorectal surgery compared with after open surgery [22, 23]. The increasing reports of laparoscopic SBO- surgery are promising [24]; however, such surgery seems to be most suitable for adhesive bands. There are several adhesion prevention substances on the market, some of which are in a form of a sheet or film, creating a localised protection area. To address the prevention of adhesions, use of fluids in the abdominal cavity might be more logical. One such fluid, icodextrin, shows a potential ability to reduce adhesions [25, 26] and has been reported as safe to use in colorectal surgery [27].

## Conclusion

In this population-based study of adhesive SBO, the incidence of surgery was 13 per 100, 000 inhabitants and year. About half of patients with SBO are elderly with co-morbidity which predisposes to postoperative complications and mortality. The mechanism of obstruction was a fibrous band in 56% and diffuse adhesions in 44%. Diffuse adhesions were related to difficult surgery and future SBO. Overall, nearly one-fifth of patients needed re-admission for recurrent SBO. Continued research for preventing SBO is desirable.

## Data Availability

The datasets used and analyzed during the current study are available from the corresponding author on reasonable request.

## Abbreviations

SBO: Small bowel obstruction
ASA: American Society of Anesthesiologist’s
ICU: Intensive care unit
CT: Computerized tomography
Dnr: Register number
COPD: Chronic obstructive pulmonary disease
MD: Missing diagnosis
